# Age of red blood cells and transfusion in critically ill patients

**DOI:** 10.1186/2110-5820-3-2

**Published:** 2013-01-15

**Authors:** Cécile Aubron, Alistair Nichol, D Jamie Cooper, Rinaldo Bellomo

**Affiliations:** 1 ANZIC Research Center, School of Public Health & Preventive Medicine, Monash University, The Alfred Center, 99 Commercial Road, Melbourne, VIC, 3004, Australia

**Keywords:** Age of red blood cells, Storage lesion, Critically ill patients, Outcome, Transfusion, Anemia, Trauma, Cardiac surgery, Mortality, Cytokines

## Abstract

Red blood cells (RBC) storage facilitates the supply of RBC to meet the clinical demand for transfusion and to avoid wastage. However, RBC storage is associated with adverse changes in erythrocytes and their preservation medium. These changes are responsible for functional alterations and for the accumulation of potentially injurious bioreactive substances. They also may have clinically harmful effects especially in critically ill patients. The clinical consequences of storage lesions, however, remain a matter of persistent controversy. Multiple retrospective, observational, and single-center studies have reported heterogeneous and conflicting findings about the effect of blood storage duration on morbidity and/or mortality in trauma, cardiac surgery, and intensive care unit patients. Describing the details of this controversy, this review not only summarizes the current literature but also highlights the equipoise that currently exists with regard to the use of short versus current standard (extended) storage duration red cells in critically ill patients and supports the need for large, randomized, controlled trials evaluating the clinical impact of transfusing fresh (short duration of storage) versus older (extended duration of storage) red cells in critically ill patients.

## Introduction

Anemia is common in critically ill patients: up to 90% of patients will be anemic by day 3 of their intensive care unit (ICU) stay [1,2]. Red blood cells (RBC) transfusion rates in critically ill patients are reported between 20% and 40% in ICU [2-4], with a mean of 2 to 5 RBC units transfused per patient [3,4]. Such anemia of critical illness has been associated with a poor prognosis even in the absence of ischemic heart disease [3,5,6]. This association supports the value of RBC transfusion in critically ill patients. Nonetheless, although potentially life-saving for individual patients, RBC transfusion also has been associated with an increased risk of morbidity and/or mortality in critically ill, surgical, and trauma populations [7,8]. In this setting, studies have increasingly focused on the possible deleterious role played by RBC storage duration (so-called age of red cells) [9-12]. In particular, they have raised concerns that prolonged RBC storage may lead to harm once such “older” red cells are transfused into ICU patients.

To avoid wasting RBC units and improve the provision of blood stock, standard practice worldwide consists of transfusing the oldest compatible and available RBC unit. In addition, RBC can be stored up to 42 days maximizing their availability and the likelihood that red cells older than 2 weeks will be transfused into critically ill patients. This RBC storage duration usually up to 42 days has been defined on the bases of 1) a percentage of RBC still present in the circulation 24 hours after transfusion higher than 75%, and 2) hemolysis <1% at the end of the storage period [13]. Despite improved preservation methods, “storage lesions” occur in such cells, because in a way that increases over time erythrocytes develop important biochemical and structural derangements that affect their function and possibly their safety [10,14].

## Current practice and concerns with “age of red cells” for ICU patients

The mean age of blood transfused in ICU patients varies from 16 to 21 days and is very similar throughout the world [2,3]. A large range of adverse effects related to RBCs storage have been reported in critically ill patients when RBC stored for 2 to 4 weeks are transfused. These include increased mortality [15-22], nosocomial infections [18,22-27], multiple organ failure [18,28], renal failure [18,22] deep vein thrombosis [20], increase in ICU [15] and hospital length of stay (LOS) [29,30], and in mechanical ventilation duration [18]. Nonetheless, most clinical studies in this area have been *observational* in nature, *retrospective* in design, *small* in size, and *subject to bias*, leaving this issue unresolved for more than 20 years.

Given the above concerns, further research to determine the effect of storage lesion (age of red cells) on clinical outcome in critically ill patients seems important. If fresh blood is associated with a decrease of mortality in critically ill patients, changes in transfusion policies will be applied. Considering the cost and the potential consequences on blood supply of delivery of fresh blood instead old blood, pivotal trials are required to answer definitively whether age of blood impacts the mortality and or morbidity of critically ill patients.

This review will consider: 1) the nature of storage lesions and why critically ill patients may be especially susceptible to the adverse effects of prolonged RBC storage; 2) the experimental studies that have explored the impact of blood storage on tissue oxygenation parameters; 3) the clinical studies of the effect of prolonged storage in adult trauma, postcardiac surgery, or ICU patients; and 4) the need for randomized, controlled studies in this field.

## RBC storage lesions

**Figure 1:**
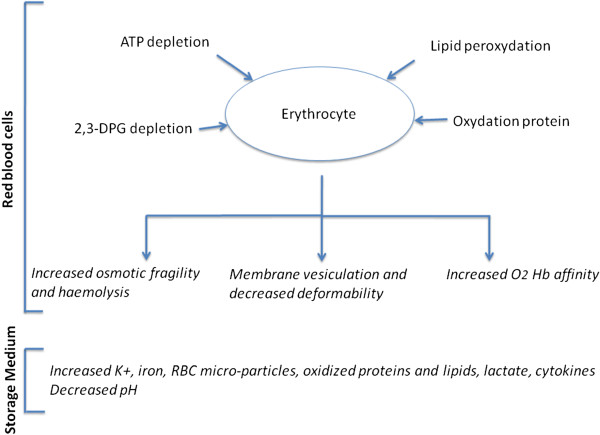
**Changes occurring in red blood cells and storage medium over the storage time. ***ATP* adenosine triphosphate; *2,3-DPG* 2,3-diphosphoglycerate; *RBC* red blood cells.

During storage, RBC and their preservative medium suffer metabolic, biochemical, and molecular changes commonly referred to as “storage lesion” (Figure 1; Table 1) [10,31]. Structural RBC changes include depletion of adenosine triphosphate (ATP) and of 2,3-diphosphoglycerate (2,3-DPG); membrane phospholipid vesiculation; protein oxidation; and lipid peroxidation of the cell membrane [10,31,32]. Over time, RBC shape changes with increased osmotic fragility and loss of deformability [31,33]. Decreased membrane flexibility may compromise the effect of RBC on microcirculatory flow and participates in increasing red cell-endothelial cell interaction, with activation of inflammatory pathways. Furthermore, bioreactive substances accumulate in storage medium. These include 1) lipids that prime recipient neutrophils and have been implicated in transfusion-related acute lung injury [34]; 2) cytokines; and 3) free iron from hemolysis [35].

**Table 1:** **Main biochemical changes in RBC storage medium and in loss of RBC deformability over the storage period **[ [10,67]]

**Variables**	**Length of storage**			
	Day 1	Days 7	Days 15	Day 42
K (mmol/L)	3.9 ± 0.6	13.6 ± 1.7	24.5 ± 2.1	46.6 ± 4.1
pH	6.8 ± 0.03	6.74 ± 0.03	6.64 ± 0.02	6.37 ± 0.04
Lactate (mmol/L)	3.6 ± 0.4	7.8 ± 0.7	17.2 ± 2.5	34.5 ± 4.4
Iron (μmol/L)	3.8 ± 0.9	6.8 ± 2.9	7.6 ± 1.6	14.2 ± 2.9
Free hemoglobin (g/L)	1.3 ± 0.5	1.5 ± 0.8	1.7 ± 0.5	3.0 ± 2.1
Percentage of irreversible deformed RBCs	-	8.4 ± 1.6	14.7 ± 2.6	29.9 ± 4.0

Recently, a study conducted in healthy volunteers reported the presence of higher extravascular hemolysis after older RBC transfusion (storage of 40–42 days) compared with fresh blood (storage of 3–7 days) illustrating the possible harmful effect of iron delivery [36]. This RBC “storage lesion” also alters oxygen delivery (because of the higher affinity of hemoglobin to oxygen secondary to a decrease in 2,3-DPG) and pH and, in addition, it increases cell lysis [10]. Free hemoglobin interacts with nitric oxide (NO) leading to endothelial dysfunction and contributing to intravascular thrombosis, vasoconstriction, and leukocyte adhesion [37]. Finally, white blood cells present in the transfused RBC can increase hemolysis and potassium release, liberate oxygen radical, and increase erythrocyte alterations [14,31].

## ICU patients may be especially susceptible to transfusion of older RBC

The severity of illness of blood transfusion recipients may increase their susceptibility to the deleterious effects of RBC storage in several ways, making these concerns particularly relevant to critically ill patients. First, critically ill patients frequently have disease states that lead to impaired microcirculatory blood flow [38]. Second, their neutrophils may be primed by a trigger event (e.g., sepsis or trauma) and subsequent exposure to bioreactive substances of RBC unit may initiate enhanced activation of adherent leucocytes [39]. This hypothetical “two-hit” model was supported by a study that compared the transfusion of fresh or aged RBC into healthy rats with lipopolysaccharide pretreated rats. Transfusion of aged erythrocytes caused mild pulmonary inflammation but no coagulopathy in healthy rats, while it augmented lung injury by inducing coagulopathy in lipopolysaccharide pretreated rats. This difference was not found with the transfusion of fresh blood [39]. ICU patients are likely to undergo invasive mechanical ventilation (MV). Experimental data report an increase of transfusion related acute lung injury in mice receiving MV compared with those without MV. MV, particularly with injurious ventilator setting, induces lung injury and increase susceptibility of TRALI [40].

Finally, a “dose-effect” may exist and, because critically ill patients are especially susceptible to receiving multiple transfusions, this effect may increase their risk of developing adverse events [20,41]. In addition, receiving multiple transfusions will increase the risk that at least one of the RBC units transfused will be “old.” In a retrospective study, Weinberg et al. found an independent increase in the risk of death when trauma patients received 6 or more RBC units with at least one RBC stored for ≥14 days [16]. The same authors, in 1,647 trauma patients transfused within the first 24 hours postinjury, reported a higher independent risk of death only in patients who received 3 or more units stored for ≥14 days (RR = 1.57, 95% CI 1.14-2.15, *p* = 0.01) [41].

## RBC storage and tissue oxygenation parameters

In critically ill patients, RBC transfusion is commonly used to restore or increase tissue oxygenation. Different surrogate markers of tissue oxygenation have been used to explore the relationship between duration of RBC storage and their efficacy in restoring optimal tissue oxygenation. In 1993, Marik et al. were the first to report a harmful effect of duration of RBC storage on systemic and tissue oxygenation. In 23 mechanically ventilated septic patients, they prospectively demonstrated an inverse association between change in gastric intramucosal pH (reflecting oxygen uptake) and age of blood [42]. Nonetheless, this was a post-hoc analysis and such a relationship was not confirmed in three subsequent studies [43-45]. One of these negative studies was a double-blind, randomized trial that compared the effect of RBC transfusion on tonometric indexes when RBC units were stored less than 5 days versus more than 20 days. This study did not show any difference between the study groups. Furthermore, there was no benefit from transfusing RBC whatever the age of blood possibly because of the defined transfusion target (80–90 g/L) or because the poor sensitivity of the tonometric technique [44]. More recently, Sakr et al. did not find any impact of storage time on sublingual microvascular perfusion in a prospective, single-center, observational study conducted in 35 patients with severe sepsis and septic shock [45]. In the setting of severe traumatic brain injury, the impact of RBC storage duration on cerebral oxygenation assessed by brain tissue oxygen pressure (PtiO_2_) was recently studied in an observational prospective study [46]. RBC stored for more than 19 days were unable to increase brain oxygenation, whereas fresh blood (<19 days) was effective [46]. In light of the contradictory findings of these five *in vivo* studies, it remains controversial whether storage lesions affect the ability of RBC to modulate tissue oxygenation.

## RBC storage and clinical outcome - Review

We searched with the PubMed database studies comparing clinical outcomes of critically ill patients receiving fresh or old blood. Excluding non-English language reports and studies conducted in pediatric patients, we identified 32 studies that examined the clinical effect of blood storage in trauma patients, ICU patients, and patients undergoing cardiac surgery or those with acute heart disease [15-30,41,47-59]. Eighteen of these studies reported a deleterious effect of increasing duration of RBC storage on clinically relevant outcomes [15-30,41] (Table 2), whereas 14 of these studies did not demonstrate any effect of prolonged RBC storage (Table 3) [47-59].

**Table 2:** Studies reporting a clinically harmful effect of prolonged RBC storage

**Authors**	**Year**	**Setting**	**N**	**Study design**	**Main confounders used for adjustment**	**Leukodepletion**	**Outcome and main results**
Purdy et al. [16]	1997	ICU, severe sepsis or septic shock	31	Retrospective single-center	No	No	Median of RBC storage was lower in survivors (17 days) than in nonsurvivors (median 25 days) (*p* < 0.0001)
Zallen et al. [28]	1999	Trauma, ≥6 RBCs units in the first 12 hours post injury	63	Retrospective single-center	Patient age, serum lactate, base deficit	No	Mean age of RBC >14 days associated with MOF (OR 1.16, CI 95%, 1.01-1.34, *p* = 0.03)
Vamvakas et al. [23]	1999	Post-CABG	416	Retrospective single-center	Chronic systemic illness, CABG surgery type, IABP, intubation, impaired consciousness, patient age, bypass time, chest tube drainage, admission WBC count	No	Oldest blood was associated with a higher risk of pneumonia and/or wound infection compared with fresh blood (median of the mean age of the oldest and second oldest RBC units = 21.6 (range: 4–41) days vs. 13 (range: 2–39) days, *p* = 0.0002)
Offner et al. [26]	2002	Trauma, ≥ 6 RBC units in the first 12 hours postinjury	61	Prospective single-center observational	Patient age, ISS, gender, mechanism of injury	No	Risk of major infectious complications increased with the number of RBC units >14 days (OR = 1.13, 95% CI, 1.01-1.26, *p* = 0.03)
Keller et al. [29]	2002	Trauma with up to 4 RBC units in the first 48 hours post injury	86	Retrospective single-center	ISS, requirement for surgery, volume of RBC, patient age	No	Association between the number of RBC >14 days and hospital LOS
Leal-Noval et al. [24]	2003	Post-CABG or valve surgery	585	Prospective single-center observational	Re-intubation, central nervous system dysfunction, Apache II score, MV duration	No	Association between older RBC (>28 days) and the risk of pneumonia (OR = 1.06, 95% CI, 1.01-1.11, *p* = 0.018) No association with mortality
Murrell et al. [30]	2005	Trauma	275	Retrospective single-center	Patient age, ISS, leukodepletion volume of RBC	95%	Association between older blood and longer ICU and hospital LOS (RR = 1.15, 95% CI, 1.11-1.2) No association with mortality
Koch et al. [18]	2008	Post-CABG or valve surgery	6002	Retrospective single-center	Baseline characteristics	Mixed	Old blood >14 days was associated with mortality, MV duration, renal failure, infections and MOF
Weinberg et al. [17]	2008	Trauma, ≥ 1 RBC unit in the first 24 hours post injury	1813	Retrospective single-center	Patient age, gender, ISS, mechanism of injury, volume of RBC, hospital LOS	Yes	Transfusion ≥6 RBC units of RBC older ≥14 days was associated with higher mortality
Weinberg et al. [22]	2008	Trauma without RBC transfusion in the first 48 hours post injury	430	Retrospective single-center	Patient age, gender, ISS, presence of thoracic injury, MV, volume of RBC	Yes	RBC ≥14 days was associated with mortality (OR = 1.12, 95% CI: 1.02 to 1.23), renal failure (OR = 1.18, 95% CI, 1.07-1.29) and pneumonia (OR = 1.10, 95% CI, 1.04-1.17) Not with ARDS
Weinberg et al. [41]	2010	Trauma, ≥1 RBC unit for the first 24 hours	1647	Retrospective single-center	Patient age, gender, ISS, mechanism of injury, volume of RBC,FFP and platelets, presence of head injury	Yes	3 or more RBC ≥14 days increased risk of death (RR = 1.57, 95% CI, 1.14-2.15, *p* = 0.01)
Spinella et al. [20]	2009	Trauma, ≥5 RBC units	202	Retrospective single-center	Patient age, cryoprecipitate, Glasgow coma score, ISS	Mixed	Association between RBC >21 days and DVT occurrence Association between RBC >28 days and mortality (OR = 4, 95% CI, 1.34-11.61)
Vandrome et al. [27]	2009	Trauma	487	Retrospective single-center	Patient age, gender, ISS, mechanism of injury and MV time	Yes	Risk of pneumonia higher in patients transfused with RBC ≥14 days (RR = 1.42, 95% CI, 1.01-2.02)
Robinson et al. [21]	2010	Post-percutaneous coronary intervention	909	Retrospective multi center	Volume of RBC, procedures details, demographic characteristics	NG	Increased in age of the youngest RBC was associated with 30-day mortality (HR = 1.02, 95% CI, 1.18-1.34, *p* < 0.001)
Eikelboom et al. [19]	2010	Acute cardiovascular disease	4933	Prospective single-center observational	Demographic characteristics, comorbidities, clinical characteristics, patient ABO group	Yes	Hospital mortality higher when the oldest RBC >31 days compared with RBC <10 days (RR = 1.48, 95% CI, 1.07-2.05)
Andreasen et al. [25]	2011	Post-CABG or valve surgery	1748	Retrospective multicenter	Place of surgery, patient age gender, BMI, preoperative Hb, diabetes, reoperation due to bleeding, use of cardiopulmonary bypass, concomitant valve surgery, comorbidities, volume of RBC and platelets units, ABO blood group	Mixed	Higher risk of severe postoperative infections (OR = 2.5, 95% CI, 1.2-4.2) in patients with RBC exclusively ≥14 days
Pettila et al. [15]	2011	ICU	757	Prospective multicenter observational	Apache III score, leukodepletion status, pre-ICU transfusion, cardiac surgery, other transfused blood components, pretransfusion Hb preceding the first transfusion, centers	80%	Oldest RBC associated with longer LOS and higher mortality
Juffermans et al. [61]	2012	Trauma	196	Retrospective single-center	ISS, head trauma, surgery, use for SDD, volume of RBC and of platelets	Yes	Patients with infections received more old blood (>14 days) than patients without infections (8 RBC units (range: 2–16) versus 4 RBC units (range: 2–8), *p* = 0.02)

*RBC* red blood cells; *ICU* intensive care unit; *LOS* length of stay; *MV* mechanical ventilation; *DVT* deep vein thrombosis; *ARDS* acute respiratory distress syndrome; *CABG* coronary artery bypass graft; *MOF* multi organ failure; *IABP* intra-aortic balloon pump; *WBC* White blood cells; *ISS* injury severity score; *APACHE* II score Acute Physiology and Chronic Health Evaluation II score; *RR* relative risk; *BMI* body mass index; *Hb* hemoglobin; *FFP* fresh frozen plasma; *SDD* selective digestive decontamination, *NG* not given.

**Table 3:** Studies reporting no clinical effect of prolonged RBC storage

**Author**	**Year**	**Setting**	**N**	**Study design**	**Adjustment for confounders**	**Leukodepletion**	**Outcome and main results**
Wasser et al. [47]	1989	Post- CABG	237	Single-center randomized Cases: RBC<12 hours; controls: RBC stored for 2 to 5 days	NA	No	No difference in bleeding and RBC transfusion requirement, nonetheless the platelets counts and thrombotest were significantly less altered in the study arm
Schulman et al. [48]	2002	Trauma, ≥2 RBC units	17	Single-center randomized pilot study “Fresh group”: RBC<11 days; “Old group”: RBC >20 days	NA	Yes	Mortality, infectious complications, respiratory failure
Vamvakas et al. [49]	2000	Post- CABG	268	Retrospective single-center	Gender, patient age, comorbidities, type of CABG, IABP, duration of anaesthesia, time on bypass, other surgery, repeated surgery, chest tube drainage volume	No	Post-operative ICU LOS, hospital LOS and MV duration
Gajic et al. [50]	2004	ICU patients with MV	181	Retrospective single-center	APACHE III score, Tidal volume, thrombocytopenia, massive transfusion	70%	Median storage duration of the oldest RBC unit = 20.3 days (range: 16–31) in absence of ALI versus 20.1 days (range: 16–27) in presence of ALI
Hebert et al. [51]	2005	ICU	57	Double-blind multicenter, randomized pilot study	Comorbidities, major diagnostic grouping, center	Yes	Composite outcome (mortality, nosocomial infections, thrombotic events, ischemic stroke)
Van de Watering et al. [52]	2006	Post- CABG RBC given during surgery and for 3 days post-surgery	2732	Retrospective single-center study Cases: RBC <18 days Controls: standard cares	Year of surgery, volume of transfusion, duration of surgery, previous CABG, number of distal anastomoses, patient age, gender, Hb at admission	No	30-day survival, hospital and ICU LOS
Taylor et al. [53]	2006	ICU	449	Prospective single-center observational	Patient age, survival probability	Mixed	Nosocomial infection, mortality, ICU and hospital LOS
Gajic et al. [54]	2007	ICU with ALI	74	Prospective single-center case–control study	Patients characteristic, transfusion factors	NG	Patients with ALI (median of average RBC storage = 22.9 days (range: 17–31) versus 22.9 days (range: 15–30) in controls (*p* = 0.801)
Yap et al. [55]	2008	Post-CABG and valve surgery, ≥2 RBC units	670	Retrospective single-center	Pre-operative risk profile, volume of RBC	<5%	Mortality, renal failure, nosocomial pneumonia, ICU LOS, MV duration
Van Buskirk et al. [56]	2010	ICU	298	Retrospective single-center	Volume of RBC, patient age, gender, severity at ICU admission, admission diagnosis	NG	Transfusion complications, change in SOFA score, ICU LOS, mortality
Katsios et al. [57]	2011	ICU	126	Prospective single-center observational	History of previous DVT, chronic dialysis, platelets transfusion, requirement of vasopressors	No	DVT
*Mckenny et al. [58]	2011	Post-cardiac surgery	1153	Retrospective single-center	Volume of RBC, baseline and patient characteristics	Yes	Early post-operative mortality, post-operative MV >72h, renal failure, infections, 30 day mortality, hospital mortality, prolonged MV, new renal failure, infectious complications and ICU LOS
Van Straten et al. [59]	2011	Post-CABG, ≤10 RBC units	3475	Retrospective single-center	Patient age, comorbidities, redo cardiac surgery, pre-operative Hb, emergency operation, perioperative MI, Re-exploration, year of operation, volume of RBC, FFP and platelets	Yes	Mortality
Kor et al. [62]	2012	ICU patients with MV	100	Double-blind randomized single-center Cases: one fresh RBC unit (<5 days) Control: one RBC unit of standard practices	NA	Yes	Change in PaO_2_/FiO_2_ ratio, in peak and plateau airway pressures, in markers of immune status and in coagulation

*NA* not applicable*; RBC* red blood cell; *ICU* intensive care unit; *LOS* length of stay; *MV* mechanical ventilation; *DVT* deep vein thrombosis; *ALI* acute lung injury; *CABG* coronary artery bypass graft, *APACHE* III score Acute Physiology and Chronic Health Evaluation III score; *Hb* hemoglobin; *MI* myocardial infarction; *FFP* fresh-frozen plasma; SOFA Sequential Organ Failure Assessment; *NG* not given.

*Blood stored up 35 days and not 42 days.

## Positive studies

### ICU patients

**Figure 2:**
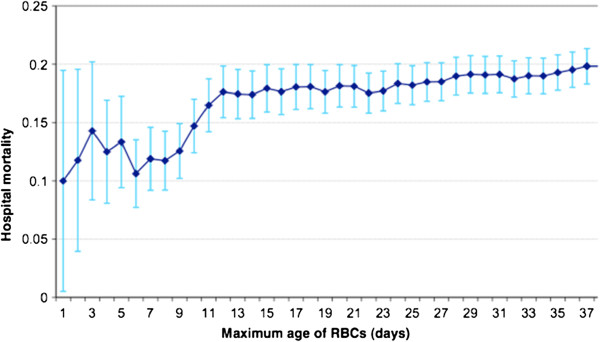
**Hospital mortality (%, 95% confidence interval) according to maximum age of red blood cells (days) from Pettila et al.**[15]**with permission.**

Two of the 18 positive studies were conducted in ICU patients [15,16]. The first was retrospective and underpowered and was conducted in 31 patients with severe sepsis or septic shock, and without adjustment for confounding factors [16]. The second was a recent, prospective, multicenter, observational study conducted in 47 Australian and New Zealand ICUs. This study enrolled 757 patients and found a significant increase in ICU LOS and in-hospital mortality rate in patients receiving the oldest blood (median of age = 17.6 days, range: 12.9-24.0) versus the freshest (median of age = 7.5 days, range: 5.7-9.0; OR = 2.01, 95% confidence interval [CI]: 1.07-3.77). This effect was seen after adjustment for severity of illness (APACHE III score), number of transfusions, pre-ICU transfusions, fresh-frozen plasma and platelet transfusions, leukodepletion status, pretransfusion hemoglobin concentration, clustering of study sites, and cardiac surgery [15]. Figure 2 illustrates the association between hospital mortality and maximum age of RBC found by these authors. However, in this observational study, patients received a mix of blood of different age (e.g., fresh and old units) and the statistical analysis could only use a surrogate of age of blood (fresh or old) received by the patients, by using the age of the *oldest RBC unit* as the unit as representative of age of red cell transfused for statistical assessment of their effect.

### Cardiovascular patients

In the setting of cardiac surgery or acute cardiovascular heart diseases, six positive studies have been published. Of these, two were prospective [19,24] and four were retrospective [18,21,23,25]. Their methods and their primary results are summarized in Table 2. Three of them have reported an increased incidence of postoperative infections in patients who were transfused with the oldest blood [23-25]. The largest study was conducted by Koch et al. and included 6,002 patients undergoing coronary artery bypass graft (CABG) and/or valve surgery [18]. Patients who received *exclusively* “oldest” blood defined by a storage duration longer than 14 days had longer duration of mechanical ventilation (9.7% vs. 5.6%, *p* < 0.001), an increased incidence of sepsis (4% vs. 2.8%, *p* = 0.01) and a higher 1-year mortality rate (11% vs. 7.4%, *p* < 0.001). Transfusion of older RBC also was independently associated with an increased risk-adjusted rate of a composite of serious adverse events (25.9% vs. 22.4%, *p* = 0.001) [18]. Despite a large sample size, this study suffered important limitations, including a retrospective and single-center design, the use of an arbitrary cutoff point to define fresh and old blood, and the absence of adjustment for some important confounding factors [60]. Two other reports, however, considered patients with heart disease also found an impact of RBC storage on mortality [19,21]. One, a retrospective multicenter study reported that the age of the youngest RBC transfused within 10 days of percutaneous coronary intervention was significantly associated with 30-day mortality after adjustment for confounders (HR = 1.02, 95% CI 1.18-1.34, *p* < 0.001) [21]. The other found a linear relationship between the age of blood and mortality in 4,933 cardiovascular disease patients in acute care facilities [19].

### Trauma patients

In trauma patients, ten studies have reported an impact of the storage duration on mortality and/or morbidity [17,20,22,26-30,41]. All were retrospective, single-center studies. Trauma patients transfused with blood older than 14 days appeared to have a higher risk of developing postinjury multiorgan failure [28], infectious complications [22,26,27,61], renal dysfunction [22], greater LOS [29], and higher mortality [17,22,41]. Regardless of the arbitrary cutoff of 14 days to define fresh and old blood, other studies have found an association between blood storage duration and hospital LOS [30], occurrence of deep vein thrombosis [20], and mortality [20,30]. Trauma patients are more likely to receive a “massive” transfusion and, as already mentioned, a dose effect of oldest blood may exist [17,20,27,41].

Despite these positive observational studies, the evidence for a harmful effect of blood storage remains uncertain. This is because few of these reports adjusted for key confounding factors, including leukodepletion, volume of RBC transfused, the year of transfusion, and ABO type, and very few analyzed RBC age as a continuous variable [60]. None adjusted for all key confounders.

### Negative studies

In contrast to the above-mentioned investigations, 14 studies did not find any relationship between blood storage duration and mortality, ICU and hospital LOS, duration of mechanical ventilation, acute lung injury, nosocomial infection, renal failure, or deep vein thrombosis occurrence (Table 3) [47-59,62].

### ICU patients

One of the seven studies conducted in ICU patients was a double-blind, multicenter, randomized pilot study that enrolled 57 patients and compared a composite outcome, including mortality, between patients who received only blood of less than 8 days of age versus standard care. The small sample size (n = 57) likely contributed to an inconclusive result [51]. Taylor et al. reported an independent increase of nosocomial infection after blood transfusion in a prospective, observational study of 449 ICU patients but did not find any effect of age of RBC [53]. Three reports have especially studied the impact of RBC age on acute lung injury (ALI) or short-term pulmonary function in critically ill patients and did not find any difference [50,54,62]. Similarly, no effect of age of blood on deep vein thrombosis, ICU LOS, and mortality was found in two recent studies of ICU patients [56,57]. All of these studies were small and likely underpowered.

### Cardiovascular patients

In postcardiac surgery patients, one of the six negative studies conducted more than 20 years ago was a single-center, randomized, controlled, blind trial that compared post-CABG bleeding in 237 patients who received either 2 units of freshly collected whole blood (fresher than 12 hours) followed by blood stored between 2 and 5 days or blood aged between 2 and 5 days. In this nonpragmatic study, there was no difference for bleeding and RBC transfusion requirements [47].

All the other negative, postcardiac surgery studies were retrospective and single center in design. Nonetheless, some had a large sample size [52,58,59]. For example, in a 2,732 patient cohort, Van de Watering et al. did not show any difference in 30-day survival, ICU, and hospital LOS between patients receiving fresh blood versus old blood, whatever the criteria to estimate the storage duration (only blood < or > 18 days, the mean RBC storage time for each patient, the storage time of the youngest RBC transfused per patient, and the storage time of the oldest RBC transfused per patient) [52]. Similar results were reported by Yap et al. who did not find any association between RBC storage duration estimated by the mean age of RBC per patient, the oldest RBC unit and RBC stored longer than 30 days, and early postoperative mortality or morbidity in 670 post cardiac surgery patients [55]. Van Straten et al. also reported no association between the risk of early and late mortality and the age or the number of RBC units transfused during or within the 5 first days post-CABG [59].

### Trauma patients

The only negative study in trauma patients was an underpowered (n = 17) single-center, randomized trial that was conducted between 2000 and 2001. The study design required 15 compatible RBC units of both study arms to be available at randomization, which impacted the study feasibility [48].

### Need for randomized, controlled trials

As outlined earlier, studies that evaluated the clinical impact of RBC storage in critically ill patients were 1) heterogeneous in outcomes, study design, population, threshold to differentiate fresh and old blood, and blood characteristics (leukodepleted or not, different storage medium, or storage medium not documented) and 2) often of insufficient quality in methodology (retrospective, observational, small sample size, no or limited inclusion of key confounding factors, and arbitrary cutoff for age of blood).

Their inherent limitations do not allow confirmation of the potential impact of duration of RBC storage on adverse effects or justify a change in current clinical transfusion practice. Two of the four randomized, controlled trials (RCTs) that evaluated clinical outcomes were very small and underpowered [48,51]. The third had a protocol based on the hypothesis that extremely fresh blood (<12 hours) may contain functionally active platelets and coagulation factors and decrease postsurgery bleeding [47], and the most recent RCT only focused on the age and effect of a single RBC unit with mainly pulmonary complications as outcome [62].

**Figure 3:**
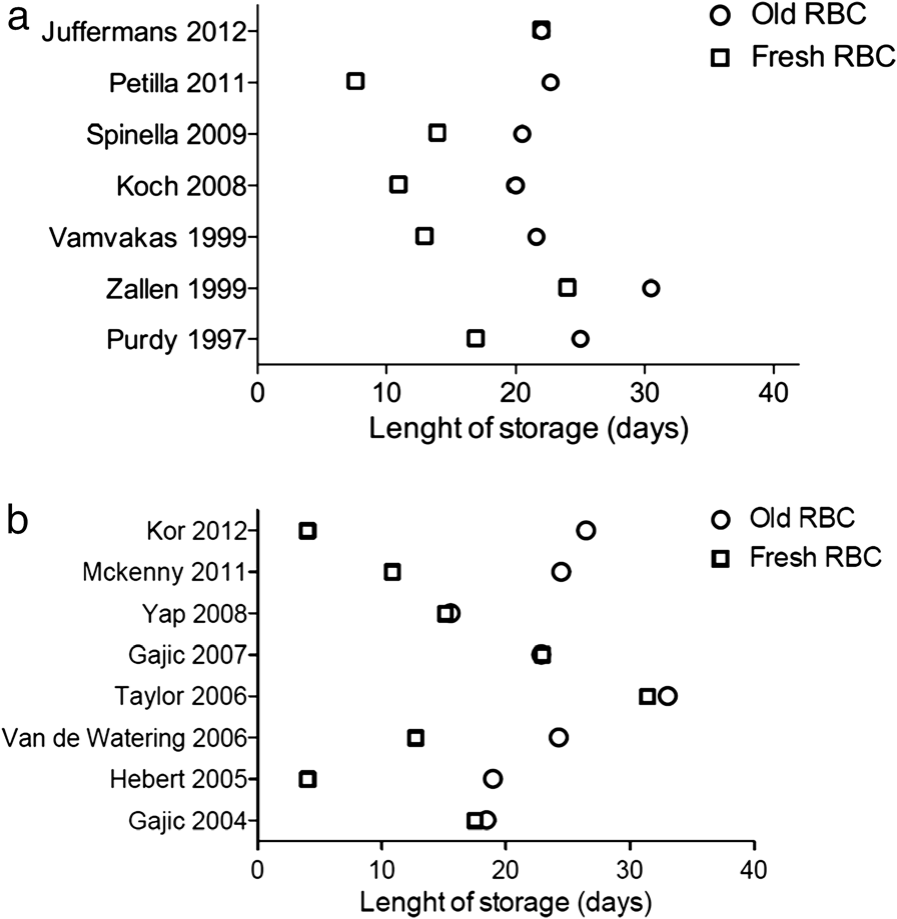
**Length of blood storage for positive (a**[15,16,18,23,27,28,61]**) and negative (b,**[50-55,58,62]**) studies.** The age of blood is expressed as the mean age or the median age of all RBC units, or in some studies as the median of the oldest (or oldest and second oldest) RBC units. The figures give the age of blood for each study comparing the primary study outcome. For instance, if the study outcome is TRALI, the figure shows the age of blood in the group of patients with TRALI and in the group of patients without TRALI.

Previous systematic reviews [9-11,13] and a meta-analysis conducted in critically ill patients [63] have been inconclusive. Recently, in a meta-analysis, including 21 studies, Wang et al. concluded that older stored blood was associated with an estimated OR for death of 1.16 (95% CI 1.07-1.24). However, some studies did not adjust the risk of death for important confounders , making their conclusions open to challenge [12]. In addition, definition of fresh and old blood remains based on observational studies and therefore unknown because of the heterogeneity of their results (Figures 3a and b).

The above observations make a large, multicenter, RCT in critically ill patients of the greatest priority [64]. In this regard, several such multicenter RCTs are currently underway in adult critically ill patients (Table 4) [65].

**Table 4:** Multicenter, randomized, clinical trials about blood storage in critically ill adults

**Authors or study name**	**Population**	**Sample size**	**Case criteria**	**Controlled criteria**	**Outcome**	**Status**
Hebert et al. [51]	ICU	57	<8 days	Standard practices	Composite outcome* (pilot study)	Achieved
Aubron et al. [67]	ICU	51	Freshest compatible available RBC	Standard practices	Feasibility (pilot study)	Achieved
**RECESS (NCT00991341)	Post cardiac surgery	1434	≤10 days	≥21 days	Change in MODS	In progress
**ABLE (ISRCTN44878718)	ICU	2510	<8 days	Standard practices	90-day mortality	In progress
**TRANSFUSE (ACTRN12612000453886)	ICU excluding postcardiac surgery	5000	Freshest compatible available RBC	Standard practices	90-day mortality	In progress

RECESS, Red Cell Storage Duration Study; ABLE, Age of Blood Evaluation; TRANSFUSE, STandaRd Issue TrANsfusion versuS Fresher red blood cell Use in intenSive carE; MODS multiorgan dysfunction score; **Indicates the trial is in progress.

*Included hospital mortality, serious nosocomial infections, thrombotic events with myocardial infarction and acute ischemic stroke.

The first such trial is the RECESS (Red Cell Storage Duration Study [RECESS]: NCT00991341) study. RECESS will randomize 1,434 cardiac surgical patients to receive either RBC stored 10 days or less or 21 days or more. However, its results could not be generalizable to any heterogeneous ICU population.

The Canadian ABLE study (Age of Blood Evaluation [ABLE] trial of the resuscitation of critically ill patients: ISRCTN44878718) is currently being conducted in ICU patients. It will enroll a total of 2,510 patients in Canada, France, and the United Kingdom [66]. The ABLE study will compare 90-day mortality between patients transfused with fresh RBC (defined as a storage duration <8 days) and patients transfused in accordance with standard practices [66]. Potential limitations of ABLE include 1) a sample size based on an estimated 25% relative risk reduction in the primary endpoint and 2) a design (fresh blood always fresher than 8 days) that seems unlikely to be reproducible always in future clinical practice.

The TRANSFUSE trial (ACTRN12612000453886) commences in 2012 in Australia and New Zealand. TRANSFUSE is a large (5,000 patients) pivotal, multicenter, randomized, controlled trial in critically ill patients to determine whether, compared with standard care, transfusion of the freshest available RBC decreases patient mortality. Completion is expected in 2015 and its findings are likely to guide blood transfusion policy in ICU patients.

## Conclusions

Blood transfusion is a common therapeutic intervention in critically ill patients. Much scientific evidence, however, supports the occurrence of alterations in red cells over their storage time, and observational studies suggest that transfusion of older RBC may have important adverse clinical consequences, including mortality in this population. Nonetheless, making structural changes in transfusion policy to deliver only fresh red cells to critically ill patients would have far-reaching logistics. The possibility that a clinically significant risk of older RBC transfusion exists and the possible benefits of making adjustments to transfusion policy can only be resolved by supporting and completing the current, pivotal, multicenter, double-blind RCTs. Until such trials are reported, any clinical practice change is premature.

## Abbreviations

ICU: Intensive care unit; RBC: Red blood cells; LOS: Length of stay; ATP: Adenosine triphosphate; 2,3-DPG: 2,3-diphosphoglycerate; APACHE III: Acute Physiology Age Chronic Health Evaluation; CABG: Coronary artery bypass graft; RCT: Randomized controlled trials; ALI: Acute lung injury.

## Competing interests

The authors declare they have no competing interests.

## Authors’ contribution

CA and RB conceived the review. CA executed the necessary search and wrote the first draft. RB, AN, and JC reviewed and modified the draft. CA and RB reviewed and completed the final version. All authors read and approved the final manuscript.
